# Deep learning pipeline for fully automated myocardial infarct segmentation from clinical cardiac MR scans

**DOI:** 10.1093/radadv/umaf023

**Published:** 2025-07-18

**Authors:** Matthias Schwab, Mathias Pamminger, Christian Kremser, Markus Haltmeier, Agnes Mayr

**Affiliations:** Department of Radiology, Medical University of Innsbruck, 6020 Innsbruck, Austria; Department of Radiology, Medical University of Innsbruck, 6020 Innsbruck, Austria; Department of Radiology, Medical University of Innsbruck, 6020 Innsbruck, Austria; Department of Mathematics, University of Innsbruck, 6020 Innsbruck, Austria; Department of Radiology, Medical University of Innsbruck, 6020 Innsbruck, Austria

**Keywords:** late gadolinium enhancement, segmentation, deep learning, infarction

## Abstract

**Background:**

Artificial intelligence (AI) has demonstrated promise in cardiovascular magnetic resonance (CMR) imaging, particularly in myocardial infarct segmentation, where it may help reduce variability and workload in clinical practice.

**Purpose:**

To develop and evaluate a deep learning-based model that performs myocardial infarct segmentation in a fully automated way.

**Materials and Methods:**

For this retrospective study, a cascaded framework of 2- and 3-dimensional convolutional neural networks (CNNs), specialized in identifying ischemic myocardial scars on late gadolinium enhancement (LGE) CMR images, was trained on an in-house training dataset of 144 examinations acquired using a 1.5 Tesla Siemens scanner collected between 2006 and 2022. On a separate test dataset from the same institution, comprising images from 152 examinations, a quantitative comparison was conducted between AI-based segmentations and manual segmentations. Further, segmentation accuracy was assessed qualitatively for both human and AI-generated contours by 2 CMR experts in a blinded experiment. Most cases underwent single human assessment, with double reading conducted only on a subset of 20 cases.

**Results:**

Excellent agreement was found between manually and automatically calculated infarct volumes (ρ_c_ = 0.9). The qualitative evaluation showed that compared to human-based measurements, the experts rated the AI-based segmentations as better representing the actual extent of infarction (*P* < 0.001) and preferred them more often (33.4% AI, 25.1% human, 41.5% equal). On the contrary, for segmentation of microvascular obstruction (MVO), manual measurements were still preferred (*P* < 0.001; 11.3% AI, 55.6% human, 33.1% equal).

**Conclusion:**

This fully automated segmentation pipeline enables the calculation of CMR infarct size without requiring any pre-processing of the input images while matching the segmentation quality of trained human observers. As automated infarct segmentation is preferred over manual segmentation, further development of this workflow toward clinical application is warranted to improve efficiencies.

**Abbreviations** 

AI = artificial intelligence; AVD = absolute volume difference; AVDR = absolute volume difference rate; CMR = cardiovascular magnetic resonance; CNNs = convolutional neural network; LGE = late gadolinium enhancement; LV = left ventricle; MVO = microvascular obstruction; PPCI = primary percutaneous coronary intervention

**Summary** 

We developed and evaluated an algorithm that performs myocardial infarct segmentation from cardiac MR images without requiring pre-processing and that outperforms trained human observers on qualitative expert judgment.

**Key Results** 

- We developed a deep learning-based algorithm specialized in fully automated infarct segmentation on late gadolinium enhancement cardiac MR images.
- The method is evaluated, both quantitatively and qualitatively, using a dataset comprising 152 MR examinations.
- In blinded qualitative assessment, experts preferred artificial intelligence (AI)-based segmentations for infarct extent more often than human ones (33.4% vs 25.1%, respectively; P < 0.001).
- The experts still favored human over AI-based segmentation for microvascular obstruction (55.6% vs 11.3%, respectively; P < 0.001).

## Introduction

Ischemic heart disease remains a leading cause of global mortality, responsible for approximately 9.1 million deaths worldwide in 2019.^1^^,^^2^ It has been demonstrated that accurately assessing infarct size and microvascular obstruction (MVO) following ST-segment elevation myocardial infarction is crucial for informed clinical decision-making and predicting major adverse cardiovascular events.^3–6^ However, obtaining these important predictors requires segmentation of late gadolinium enhancement (LGE) cardiovascular magnetic resonance (CMR) images.

Manual LGE segmentation by expert readers is time-consuming and suffers from limited reproducibility,^7^ prompting the development of deep learning-based algorithms for automatic infarct segmentation.^8–15^ However, these frameworks rely heavily on quantitative performance metrics, such as the Dice coefficient, which may not fully capture segmentation quality.^16^ While some studies have explored qualitative evaluation methods for medical image segmentation tasks,^17–23^ no qualitative assessment for artificial intelligence (AI)-generated myocardial infarct segmentation has yet been published.

The purpose of this study was 3-fold. First, to develop and evaluate a deep learning-based algorithm for accurate and fast segmentation of myocardial infarction and MVO on clinical LGE CMR images in a fully automated way, without human intervention or preprocessing. Second, to test the segmentation performance of the developed framework, by calculating quantitative metrics between AI segmentations and human-created reference standard measurements. Third, to qualitatively assess the segmentation quality for clinical use by 2 expert radiologists.

## Materials and methods

### Patient selection

This was a retrospective study of the quantitative and qualitative performance of a deep learning segmentation algorithm for myocardial infarct quantification on clinical data. All the LGE CMR images used in our study were originally acquired prospectively at a single center between 2006 and 2023 as part of the MARINA-STEMI (Magnetic Resonance Imaging In Acute ST-Elevation Myocardial Infarction) study (NCT04113356), which was approved by the local ethics committee, with all patients providing written informed consent before inclusion. Details about dataset specifics and inclusion criteria are described in Appendix S1. While several articles have been published in the last decade^24–26^ using patients from this cohort to address clinical questions, this paper is the first to take a machine learning approach to these data.

### MR image acquisition

All images in this study were acquired on 2 identical 1.5 Tesla MR scanners from the same vendor (Magnetom Avanto^Fit^, Siemens, Erlangen, Germany) 10-20 minutes after intravenous gadolinium contrast administration. Exact details of the imaging protocol have been published in Klug et al.^27^

### Training dataset

An in-house training dataset consisting of 144 LGE CMR examinations from 142 unique patients was created from data collected at the Department of Radiology. The data included images acquired at baseline (within 1 week after primary percutaneous coronary intervention, PPCI), as well as follow-up examinations at 4 months and 12 months. During training, segmentation performance was evaluated after each epoch on a hold-out evaluation dataset consisting of 33 LGE examinations (Table 1).

**Table 1.** Patient demographics of the different datasets.

	Training	Evaluation	Test
Examinations	144	33	152
Baseline	54	12	33
4FU	24	3	43
12FU	66	18	76
Age	57 ± 12 (29 − 84)	59 ± 13 (34 − 88)	61 ± 10 (42 − 86)
Sex (male/female)	118/26	24/9	126/16

Data are numbers of patients or means ± standard deviations, with ranges in parentheses. Baseline: examination within 1 week after PPCI.

Abbreviation: 4FU = 4 months follow-up examination after PPCI; 12FU = 12 months follow-up examination after PPCI; PPCI = primary percutaneous coronary intervention.

### Segmentation

**Figure 1.**
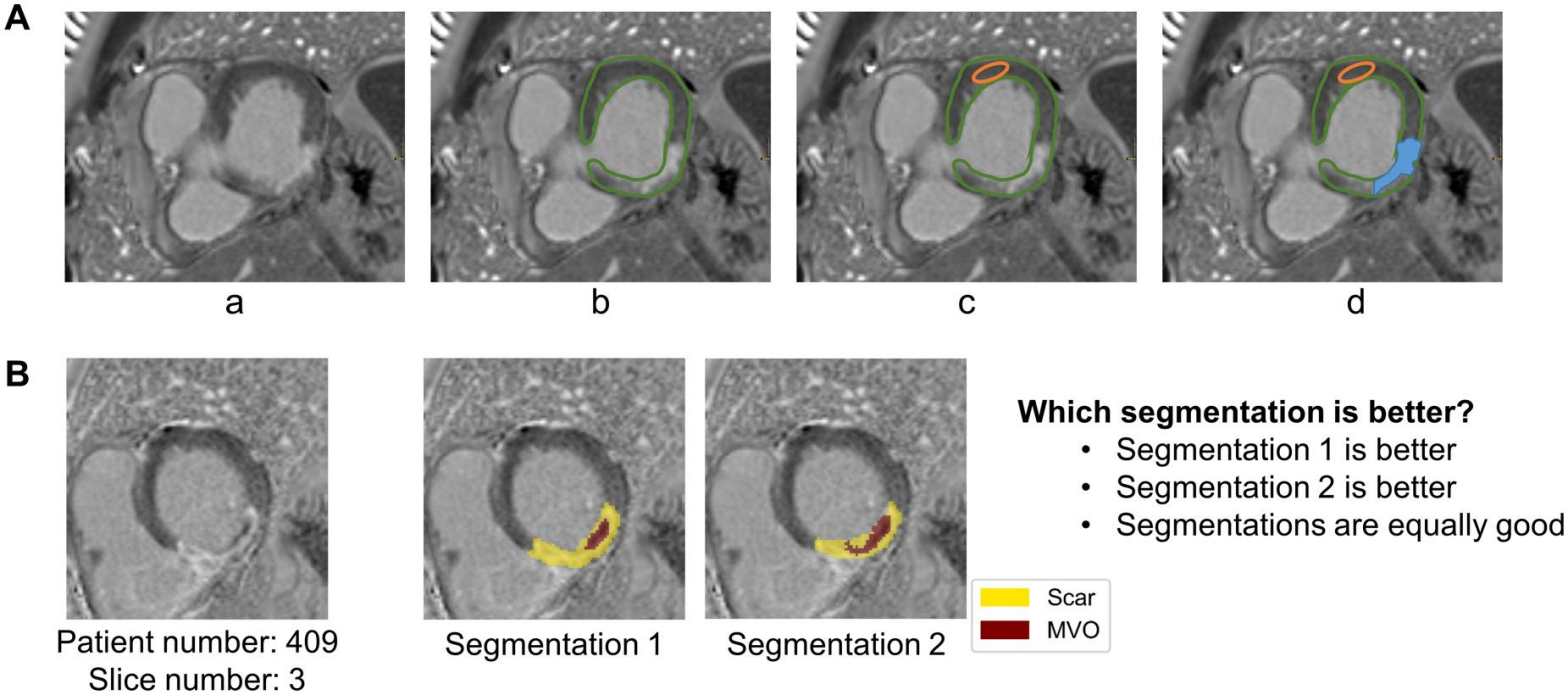
Manual segmentation procedure (A). A slice is checked for late gadolinium enhanced tissue (a). If infarction is present, epi and endocardial borders (continuous line) are drawn (b). A region of interest in the remote myocardial segment (small oval) is drawn (c). After windowing using a threshold of 5 standard deviations above the mean signal in the remote myocardium, the myocardial scar (filled area) is marked (d). Subjective comparison of human and AI-based segmentations (B). Not knowing which segmentation belongs to which method, the raters compared the 2 segmentations and reported their assessment.

Manual segmentations of the left ventricle (LV) were done by medical students serving as research assistants during their doctoral studies using the local routine diagnostic interpretation and reporting software (DeepUnity Diagnost, Dedalus Healthcare Systems Group, Germany). All manual LGE segmentations were performed according to the guidelines explained in Figure 1A. Detailed information about the manual segmentation process is provided in Appendix S2. Before performing segmentations on the actual datasets, all research assistants were trained on a separate dataset consisting of 30 patients. Raters had to achieve acceptable inter-rater reliability (κ > 0.75) before they were allowed to perform segmentations on the actual datasets.

For both the test and training datasets, manual segmentation was not all done at the same time but over the years by different members of the research group. In each of the short-axis slices of the CMR, the following 4 tissue regions were segmented if present: remote myocardium, LGE-enhanced myocardium, MVO, and blood pool. Binary segmentation masks were created from these manually defined regions for training the deep learning models. Additionally, attention was paid to marking blood within the LV outflow tract.

### Test dataset

The study analyzes the algorithm’s performance on a CMR LGE test dataset consisting of images obtained at the same institution as the training dataset. In total, images from 152 LGE CMR measurements were analyzed, including data from 121 unique patients with ST-segment elevation myocardial infarction who underwent successful PPCI. Segmentation masks for LGE-enhanced myocardium and MVO were drawn by appropriately trained research assistants as explained in the Segmentation section. Exact details about the scanners and imaging protocols used for the test, evaluation, and training datasets are described in Appendix S3.

### Deep learning pipeline

Our deep learning pipeline consists of 2 main steps: (1) extracting a stack with smaller image sizes out of the original data that still contains the entire LV, and (2) performing multiclass segmentation with a special focus on the myocardial scar on the extracted volumes.

**Figure 2.**
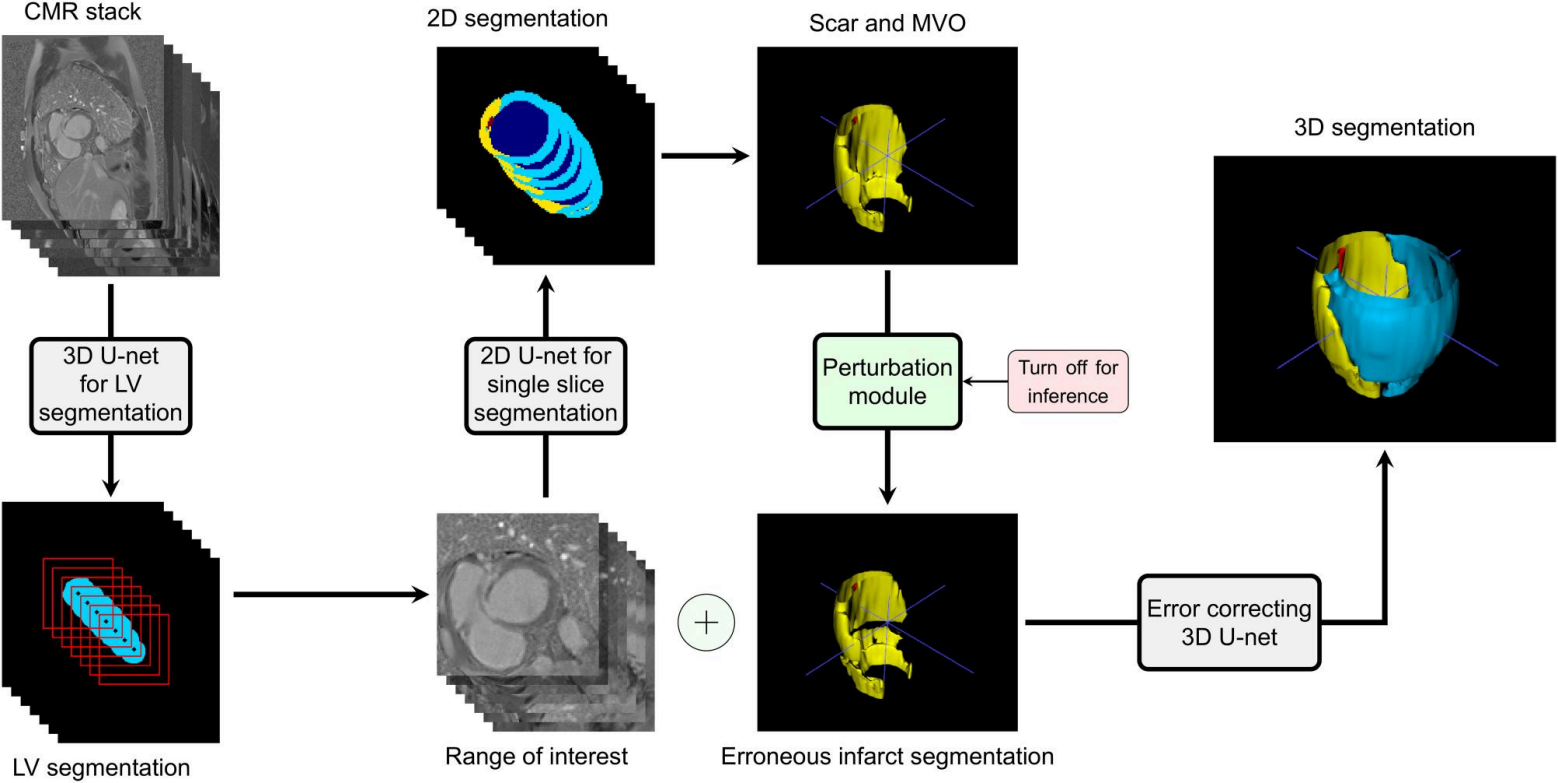
AI pipeline. First, a smaller image stack is extracted from the original data by segmenting the left ventricle. Then, an error-correcting 2D-3D cascaded framework is used to perform multiclass segmentation on the left ventricle. Abbreviations: CMR = cardiovascular magnetic resonance; LV = left ventricle; MVO = microvascular obstruction.

As shown in Figure 2, a 3D U-Net is first used to segment the LV, after which the center of mass of the middle slice segmentation is computed to extract a centered, reduced-size image stack. Next, an error-correcting 2D-3D cascaded network^28^ segments myocardial scars, leveraging both 2D and 3D data. The 2D network is trained on abundant slices, and the 3D network refines results by learning from inter-slice relationships and perturbed 2D masks. Further architectural and training details are available in the Supplementary Material (Appendices S4 and S5, Figures S1 and S2). The implementation code for the full pipeline, including all model architectures and trained weights, is publicly available at https://github.com/matthi99/FAMyoS.

### Evaluation of segmentation performance

Our evaluation of segmentation performance analysis consists of (1) a quantitative assessment of segmentation accuracy comparing AI-segmentations to manual markings and (2) a qualitative assessment of segmentation accuracy done by 2 CMR experts with 6 and 16 years of CMR experience, one of whom holds the Euro CMR level II and the ESCR diploma. For the qualitative assessment of segmentation accuracy, both manual and automatic segmentation masks were evaluated by at least one of the experts in a blinded experiment. For each patient, we randomly distributed manual and automatic segmentations into 2 groups: *segmentation 1* and *segmentation 2*. Not knowing which mask was created by humans and which by AI, the experts had to subjectively assess the segmentation quality of LGE and MVO segmentations.

On a per-slice level, they had to decide for each segmentation between different ratings (Figure S3): optimal (the segmentation was done to their full satisfaction), too big (an infarct/MVO was correctly identified, but the area marked was too big), too small (an infarct/MVO was correctly detected, but too small an area was marked), wrong tissue (areas outside the myocardium were marked as infarct/MVO), false negative (an infarct/MVO was overlooked in this slice), false positive (an area in the myocardium was falsely marked as infarct/MVO in a slice where no infarct/MVO is present), and true negative (rightfully nothing was marked in a slice where no infarct/MVO is present).

After evaluating the segmentation individually, the experts had to compare the 2 methods side by side and decide which segmentation they agreed with more. For this task, they could choose between *segmentation 1*, *segmentation 2*, or *equally good* (Figure 1B).

The data were distributed between the 2 raters in the following way: For a randomly chosen subset of 20 patients, both experts gave their ratings independently of each other. Then the agreement between their answers was evaluated, and cases where the experts disagreed were discussed in more detail. The remaining dataset was then split between the 2 experts so that each evaluated half of the remaining patients. For images in which the qualitative assessment was not entirely clear, the experts reached a consensual decision. Further details about the design of the qualitative experiments can be found in the Supplementary Material (Appendix S6, Figure S3).

### Statistical analysis

To quantify the segmentation accuracy of our method, we calculated different metrics between AI and human-generated measurements. Dice similarity coefficients (DCS) were calculated to assess the geometrical agreement between the methods. Further, clinical metrics like absolute volume difference (AVD) in mL as well as absolute volume difference rate (AVDR) with respect to the volume of the myocardium (*V*_MYO_) were calculated. The performance metrics between AI segmentations *P* and manual segmentations *G* were obtained as follows:


$$\begin{array}{l} D\mathrm{CS}=\frac{2|P\cap G|}{\left|P|+|G\right|},\\ A\mathrm{VD}=|\left|P|-|G\right||\times \text{voxelvolume},\\ \mathrm{AVDR}=\frac{\mathrm{AVD}}{V_{\mathrm{MYO}}}, \end{array}$$

where | · | denotes the cardinality of a set.

To quantify the agreement and reliability between AI and human-based infarct size measurements, we calculated the infarct volumes as a percentage of the total LV myocardial mass for both methods. Statistical analysis included a paired Wilcoxon signed rank test, Lin’s concordance correlation coefficient (*ρ*_c_),^29^ and Bland-Altman analysis.

In the qualitative analysis, relative proportions of the given answers for both human-based and AI-based segmentations were compared, and 95% confidence intervals using approximate parametric bootstrapping^30^ were calculated. A one-way chi-square test was used to identify significant differences in frequency between the experts’ assessments. Further, rater agreement was investigated by calculating confusion matrices and Cohen’s kappa coefficients (*κ*), interpreted as slight (≤ 0.20), fair (0.21-0.40), moderate (0.41-0.60), substantial (0.61-0.80), and almost perfect (>0.80) agreement.^31^

Statistical analysis was performed with Python (version 3.9) using the scipy package. For all statistical tests, a statistical significance threshold of 0.05 was used.

## Results

### Quantitative segmentation accuracy

**Figure 3.**
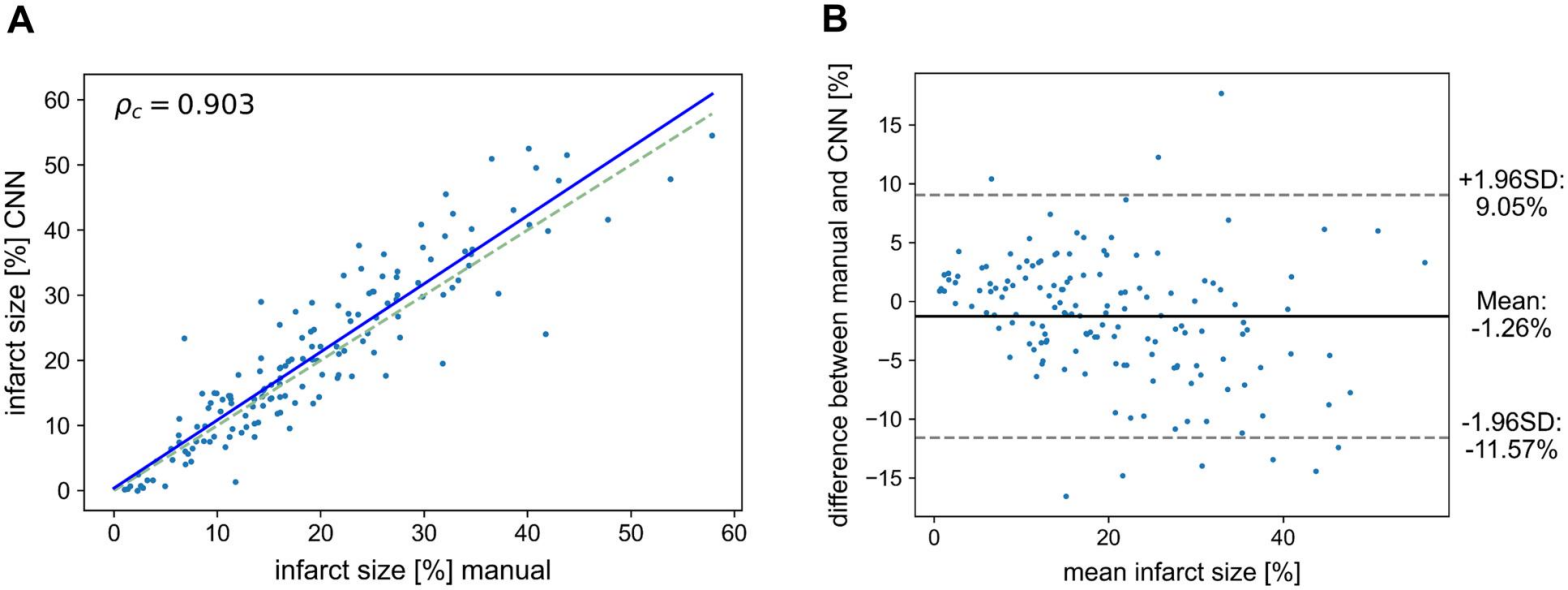
Scatter plot (A) and Bland-Altman analysis (B) of infarct size as a percentage of the total myocardial volume determined automatically and manually. In the scatter plot, the dashed line represents 100% agreement, and the solid line represents the linear regression line. Abbreviations: CNN = convolutional neural network; SD = standard deviation.

The deep learning method reached mean Dice coefficients of 64.1% for infarct segmentation and 82.2% for MVO segmentation. The mean AVD between manually and automatically calculated infarct volumes was 5.0 mL, and the mean AVDR was 4.0%. For MVO, only very small volume differences were found, with a mean AVD of 0.6 mL and a mean AVDR of 0.4%. However, MVO was present in only 15% of all the patients in the test dataset. As the Dice coefficient is undefined when both the reference and the predicted segmentation masks are empty, it was set to 1 for such cases. This resulted in optimal metric values for all the patients, where the method correctly detected no MVO. When only including patients with MVO, the mean Dice score decreased significantly to 25.0%, and similarly, the accuracy for AVD and AVDR also decreased considerably (see Table 2). Scatter plot and Bland-Altman analysis (Figure 3) showed good agreement between manually and automatically calculated infarct sizes, expressed as a percentage of the total myocardial volume. Concordance correlation was ρ_c_ = 0.90 (95% CI [0.87, 0.92]), and Bland-Altman analysis showed an average difference of −1.3% between manual and convolutional neural network (CNN) volume calculations. However, the neural network’s bias in marking slightly larger scars was statistically significant (*P < *0.01). The limits of agreement in the Bland-Altman analysis ranged from −11.6% to 9.1%.

**Table 2.** Quantitative metrics for infarct and microvascular obstruction (MVO) segmentation on the 152 cardiac MR examinations of the test dataset.

	Infarction (entire test set)	MVO (entire test set)	MVO (subgroup with MVO present)
DCS (%)	64.11 ± 18.37	85.20 ± 32.93	25.04 ± 25.28
AVD (mL)	4.97 ± 5.07	0.59 ± 2.14	3.92 ± 4.39
AVDR (%)	4.04 ± 3.5	0.43 ± 1.49	2.86 ± 2.93

The right-hand column contains the MVO results obtained exclusively for those patients in whom MVO was present (*n *= 23). Values are reported as mean ± standard deviation.

Abbreviations: AVD = absolute volume difference; AVDR = absolute volume difference rate with respect to myocardium volume; DCS = Dice similarity coefficient.

### Qualitative segmentation performance

**Figure 4.**
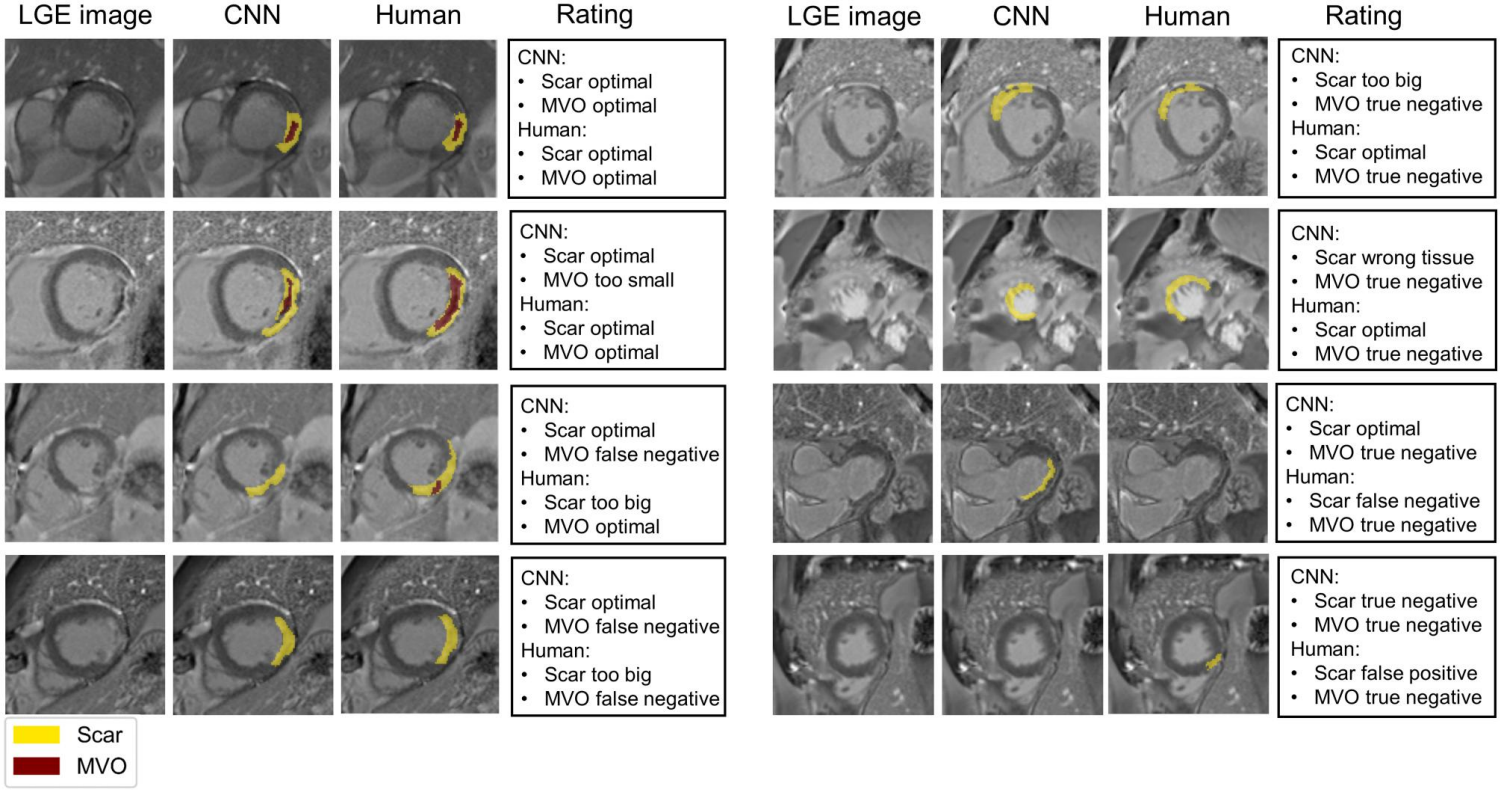
Examples of infarct segmentations, which include both optimal and faulty CNN and human-based segmentations. Expert ratings for the corresponding images are displayed for scar and MVO segmentation. Abbreviations: CNN = convolutional neural network; LGE = late gadolinium enhancement; MVO = microvascular obstruction.

**Figure 5.**
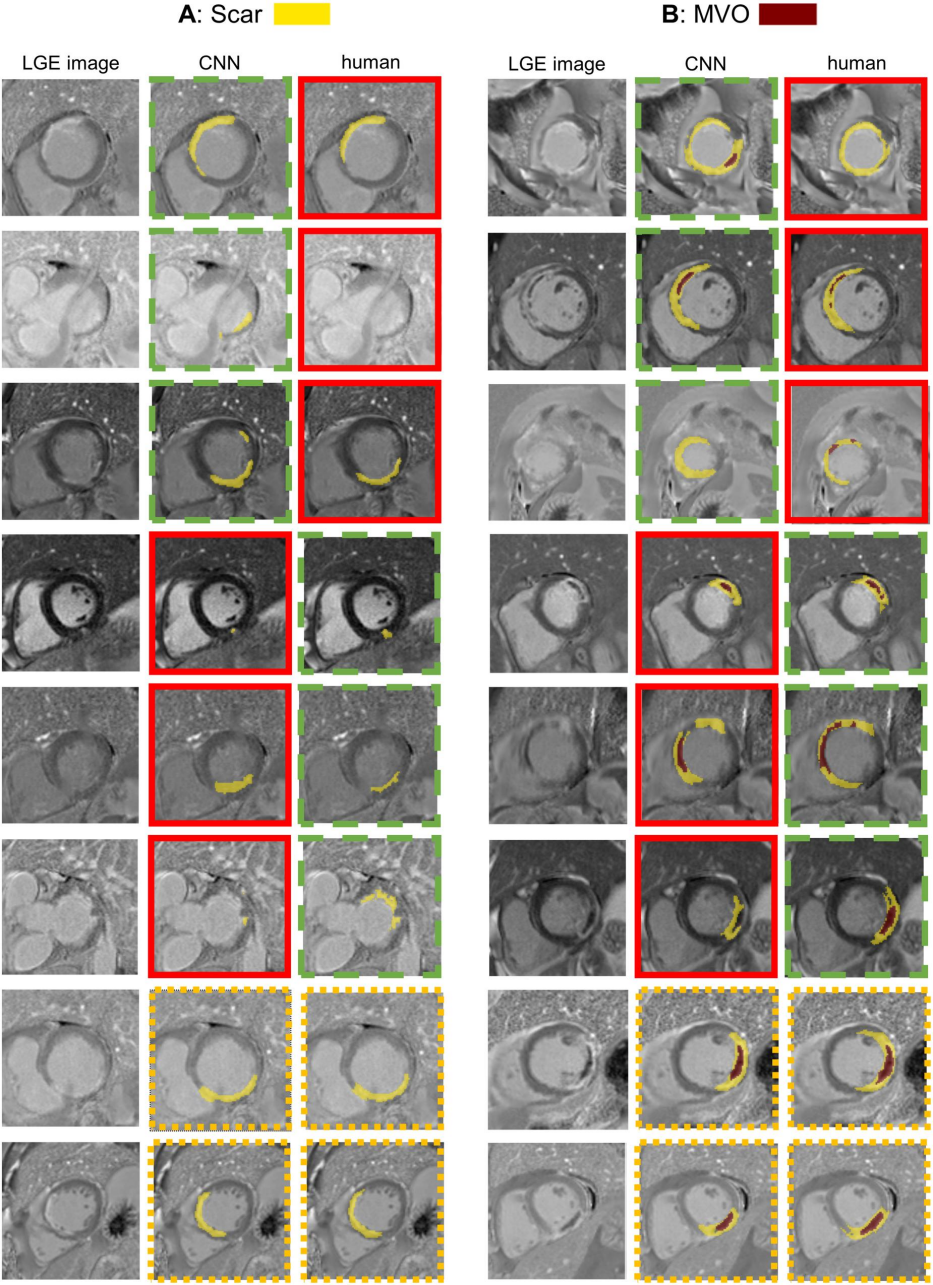
Visual comparison of human and convolutional neural network (CNN) segmentations across different preference outcomes for scar (A) and microvascular obstruction (MVO) (B) on late gadolinium enhancement (LGE) images. The preferred segmentation in each pair is highlighted with a green frame (dashed line), while the non-preferred segmentation (in cases of a clear preference) is marked with a red frame (continuous line). When both were rated equally, orange frames are used for both (dotted lines).

In total, the qualitative segmentation analysis involved ratings for LGE and MVO segmentation on 1619 pairs of CMR slices. Examples of automatically and manually created segmentation masks with corresponding expert ratings are shown in Figures 4 and 5.

Based on the experts’ validation of the segmentations, we investigated the diagnostic performances of human-based and AI-based predictions. For myocardial scars, diagnostic performance was very high, as the AI framework only missed 2 scars in the whole dataset. However, these 2 scars were tiny and could only be clearly confirmed by the CMR experts after an additional review of the functional images and previous examinations (Figure S4). For MVO detection, though, AI-based predictions showed a considerably lower sensitivity (65%) compared to humans (91%). Contingency tables and corresponding sensitivity and specificity values are shown in Table 3.

**Table 3.** Diagnostic performances of AI and humans on a per-patient level based on the expert’s blinded evaluation of the segmentations.

AI-based scar detection Sens.: 99% [95.3, 99.8] Spec.: -				Reference standard based on experts		
				Scar	No scar	Σ
AI-based MVO detection Sens.: 65% [42.7, 83.6] Spec.: 97% [92.3, 99.2]				Reference standard based on experts		
				MVO	no MVO	Σ
Human-based MVO detection Sens.: 91% [72.0, 99.0] Spec.: 99% [95.8, 100]				Reference standard based on experts		
				MVO	no MVO	Σ
Human-based scar detection Sens.: 100% [97.6, 100] Spec.: -				Reference standard based on experts		
				Scar	No scar	Σ
AI-based prediction		Scar		150	0	150
		No scar		2	0	2
		Σ		152	0	152
AI-based prediction	MVO			15	4	19
	No MVO			8	125	132
	Σ			23	129	152
Human-based prediction			MVO	21	1	22
			No MVO	2	128	130
			Σ	23	129	152
Human-based prediction		Scar		152	0	152
		No scar		0	0	0
		Σ		152	0	152

Contingency tables for AI-based and human-based scar and MVO detection. Sensitivity and specificity levels are displayed with 95% confidence intervals in brackets. Abbreviations: Sens. = sensitivity; Spec. = specificity; AI = artificial intelligence; MVO = microvascular obstruction.

Detailed results of the expert ratings for LGE and MVO segmentation are displayed in Table 4. For LGE segmentation, raters overall preferred the automatic measurements. In 33.4% of the cases, they decided to agree more with the segmentation done by the CNN, whereas in only 25.1% of the cases, manual segmentation was preferred. When excluding all cases rated as equal, a one-way chi-square test revealed that AI segmentations were preferred significantly more often (*P < *0.001). For LGE segmentation, fewer scars were overlooked (false negative) by the CNN (2.6%) compared to humans (4.2%). However, the fraction of wrongly marked infarct scars (false positives) was bigger for the CNN-generated segmentations (1.8%) compared to the human-created contours (0.8%). Total failure due to the marking of a myocardial scar in the wrong tissue has hardly ever been observed with either method (*<*1%).

**Table 4.** Expert ratings for manual and automatic late gadolinium enhancement (LGE) and microvascular obstruction (MVO) segmentations in percent with 95% confidence intervals in brackets.

Category	LGE		MVO	
	CNN (%)	Manual (%)	CNN (%)	Manual (%)
Classification:				
True negative	34.4 [32.0, 37.1]	35.3 [32.7, 37.9]	91.8 [90.6, 93.1]	92.3 [91.1, 93.5]
True positive	61.1 [58.7, 63.7]	59.6 [57.1, 62.1]	3.9 [2.7, 5.2]	5.2 [4.0, 6.4]
False negative	2.6 [0.0, 5.2]	4.2 [1.7, 6.8]	3.8 [2.6, 5.0]	2.2 [1.1, 3.4]
False positive	1.8 [0.0, 4.4]	0.8 [0.0, 3.4]	0.6 [0.0, 1.8]	0.3 [0.0, 1.5]
True positive rating:				
optimal	47.7 [45.2, 50.3]	44.9 [42.3, 47.5]	1.2 [0.0, 2.4]	4.0 [2.8, 5.2]
Too big	7.8 [5.3, 10.5]	8.5 [5.9, 11.1]	0.1 [0.0, 1.3]	0.4 [0.0, 1.6]
Too small	5.3 [2.8, 8.0]	6.1 [3.5, 8.7]	2.6 [1.4, 3.9]	0.8 [0.0, 2.0]
Wrong tissue	0.2 [0.0, 2.8]	0.2 [0.0, 2.8]	0.0 [0.0, 1.3]	0.0 [0.0, 1.2]
Segmentation preference:				
Preferred	33.4^a^ [30.2, 36.7]	25.1 [21.9, 28.4]	11.3 [3.5, 20.2]	55.5^a^ [47.9, 64.6]
Equal	41.5 [38.2, 44.8]		33.1 [25.3, 42.0]	
Segmentation preference on true positive cases:				
Preferred	30.2^b^ [26.6, 33.7]	22.6 [19.0, 26.0]	3.8 [0.0, 18.6]	87.2^a^ [70.8, 91.5]
Equal	47.3 [43.8, 50.8]		9.0 [6.2, 26.9]	

All slices were classified into true negative, true positive, false negative, and false positive (classification). For true positive segmentations, the raters had to provide more detailed feedback (true positive rating) and finally compare the 2 segmentation methods (segmentation preference). For the evaluation of segmentation preference between AI and human-created segmentation masks MR slices in which both methods correctly showed no LGE/no MVO were excluded, as the segmentations in these slices could only be evaluated as equally good. For the evaluation of segmentation preference on true positive cases only cases where LGE/MVO was indeed detected by both humans and AI were included. Statistical significance comparing preferences between CNN and manual segmentation is indicated by *asterisks*.

a *P *< 0.001.

b *P *< 0.01.

Abbreviations: CNN = convolutional neural network; LGE = late gadolinium enhancement; MVO = microvascular obstruction.

In contrast to LGE segmentation, manually created measurements remained superior to the deep learning algorithm for MVO quantification. The experts decided significantly more often (*P < *0.001) in favor of the manual MVO segmentations (55.6%) compared to the CNN-generated measurements (11.3%). The main difference between manual and automatic segmentations was in sensitivity. CNN-generated segmentation missed MVO (false negative) in quite a few of the slices (3.8%), especially when considering that MVO was only present in 7.4% of all slices. Furthermore, in slices where MVO was correctly detected, the framework tended to mark a too small area (2.6%). In contrast, 4.0% of human segmentations were rated as optimal, and only 0.8% were considered too small. However, humans also missed a substantial number of slices (false negatives) where MVO was present (2.2%).

A subanalysis including only images with true positive segmentations by both the AI and human raters showed that, even within this subset, raters tended to prefer AI segmentations for LGE and human segmentations for MVO (see Table 4).

#### Rater agreement

**Figure 6.**
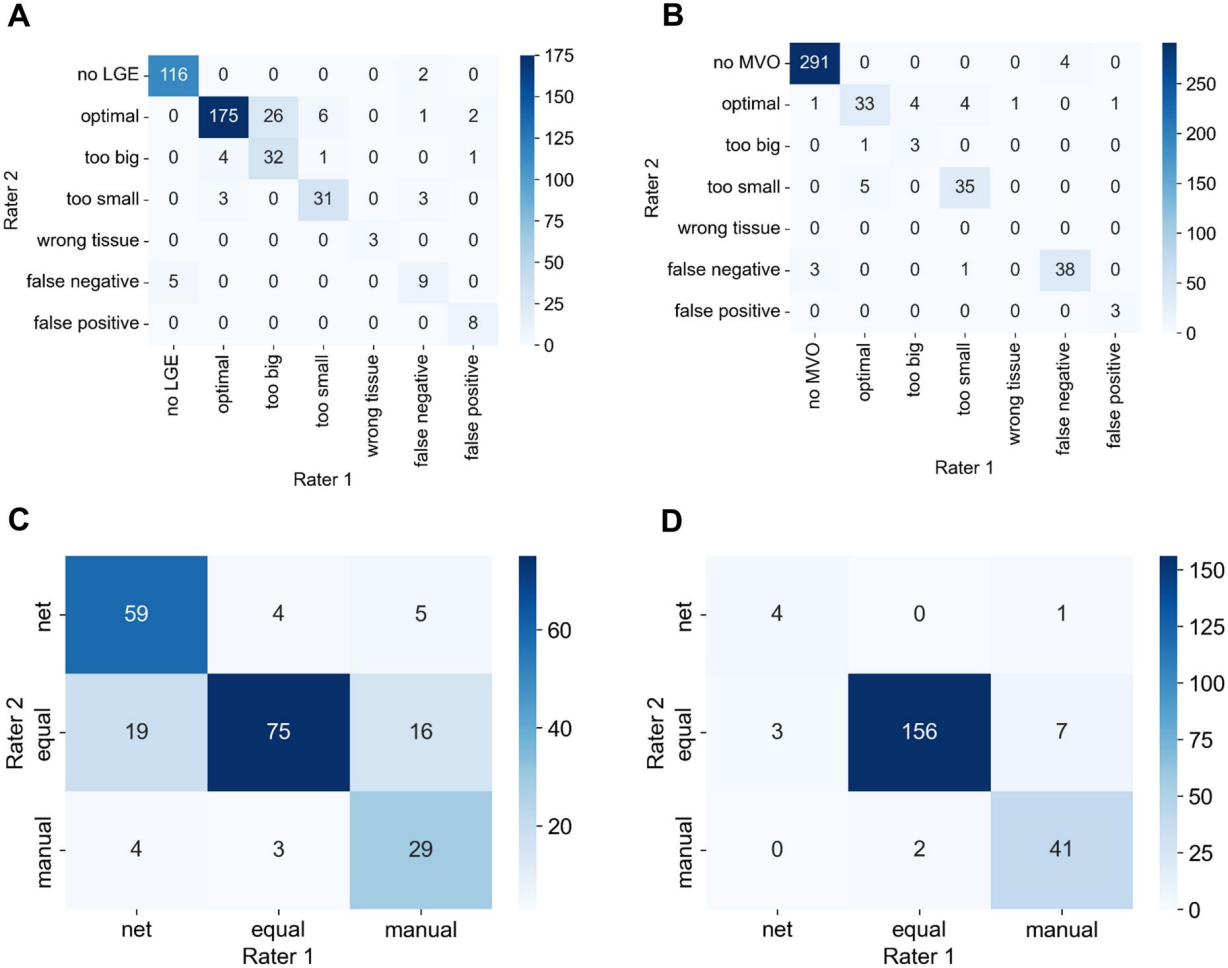
Confusion matrices on rater agreements for single image evaluation for late gadolinium enhancement (LGE) (A) and microvascular obstruction (MVO) (B) segmentations. Rater agreements for direct comparisons between the 2 methods are shown for LGE (C) and MVO (D).

In addition, to confirm the informative value of the subjective ratings of the 2 experts, we evaluated their agreement on a subset consisting of 20 patients (Figure 6). Calculating Cohen’s kappa coefficients (*κ*) revealed that the strength of agreement between the raters was almost perfect for both LGE (*κ  *=  0.82) and MVO (*κ  *=  0.88) ratings.

In rating LGE segmentation, the biggest difference between the experts was that in 26 out of the total 212 slices, rater 1 decided that the LGE segmentation was too big, while rater 2 considered it optimal. Similarly, for MVO assessments of 4 cases each, rater 1 believed that the markings were too large or too small, while rater 2 opted for optimal. In contrast, in 5 images, rater 1 voted for an optimal segmentation, while rater 2 assessed the MVO segmentation as too small. When answering the question which segmentation was better, calculating linearly weighted Cohen’s kappa revealed substantial agreement (*κ  *=  0.64) and almost perfect agreement (*κ  *=  0.83) between the raters for LGE and MVO segmentation, respectively.

## Discussion

In this work, we developed and tested a deep learning-based pipeline that enables the fully automated quantification of infarct scars and MVO from LGE CMR images. The method achieved mean Dice coefficients of 64.1% for infarct segmentation and 25.0% on a subset of MVO-positive patients. The mean AVD for infarct volumes was 5.0 mL, with a concordance correlation of 0.9. The qualitative evaluation showed AI-based segmentations were rated better for infarction compared to human segmentations (33.4% vs 25.1%, respectively; *P* < 0.001), while human measurements were preferred for MVO segmentation (55.6% human vs 11.3% AI, *P* < 0.001).

We tested the method on data from 121 unique patients, representing an increase compared to previous studies^8^^,^^10–15^ that evaluated their methods using datasets of up to 50 patients. In addition, unlike previous work, which primarily relied on quantitative metrics, we also performed a qualitative evaluation of automated myocardial infarct segmentation. This provided additional information about the types of segmentation errors made by neural networks and humans.

Although Dice scores are an imperfect metric for measuring the pure overlap of segmented areas, our results indicate that the deep learning framework still performs well for clinically relevant metrics. Therapies, risk stratification, and prognoses often rely on thresholds or ranges of infarct volume, rather than exact boundaries, making AVD and AVDR more important metrics in day-to-day clinical work. The AI model demonstrated decent performance in metrics directly relevant to clinical practice, as evidenced by AVD and AVDR, and showed reasonable diagnostic performance, as only 2 minimal scars were entirely missed by the framework in the 152 examinations. Additionally, our reported mean Dice score, as well as the values in the Bland-Altman analysis, are comparable to the results reported in inter-observer studies.^15^^,^^32^ Dice coefficients for MVO segmentation were higher than for LGE segmentation. However, largely influenced by the high number of cases without any MVO, as the Dice score massively drops when considering MVO-positive patients only. This reflects the difficulty of accurately segmenting small and irregularly shaped MVO regions.

In our qualitative evaluation, 2 experienced CMR experts significantly preferred the deep learning-based measurements in direct comparisons. We hypothesize that this is because the AI segmentations are more consistent across slices and patients, thereby avoiding the variability and subjectivity that often characterize manual annotations, especially in our clinical setting, where manual annotations were performed by different observers over a 2-year period. Furthermore, manual LGE segmentation was performed using a clinically recommended thresholding method, which is known to be influenced by the choice of the remote myocardium region, thereby contributing to the theory of larger inconsistency in manual segmentation.^33^ AI also demonstrated better sensitivity, as fewer myocardial scars were overlooked compared to human assessment on a per-slice level. This may be because human observers are potentially affected by fatigue or time constraints and therefore may overlook subtle infarcts, which are identified more reliably by the AI model.

However, these findings should be interpreted cautiously. Our study does not demonstrate that AI outperforms experts in all contexts but does so under the specific conditions tested. In contrast, for the detection and quantification of MVO, it was found that manually created measurements remained superior. Although the specificity between humans and AI was comparable, there was a significant difference in sensitivity, as many MVOs were overlooked or only partially marked by the deep-learning-based framework. The reason for this could be that MVO was only present in 25% of the patients used for training, covers only very small areas, and additionally has a very similar pixel intensity to healthy myocardium. Although MVO size is often underestimated on LGE images compared to first-pass perfusion or early-gadolinium enhancement sequences,^34^ the identical images used for both human observers and the CNN ensure comparability. Furthermore, LGE offers higher spatial and contrast resolution,^35^ enabling full coverage of the LV myocardium, which should support fair MVO detectability by both humans and the network.

Our framework had several limitations. First, our study included data from infarction patients and expert readers from a single hospital and thereby lacked external validation and prevented the assessment of potential false positives in healthy individuals. Second, all the images were acquired at 1.5 Tesla with scanners from a single vendor. Thus, this study cannot demonstrate the generalizability or reliability of our framework with other scanner types and imaging protocols. Future research should focus on clinical validation through multi-center studies with heterogeneous populations and broader vendor and reader inclusion. When considering the results of the qualitative evaluation, one could argue that even the reference standard we used for training has errors, which would mark a clear limitation. However, this could be an inherent problem of infarct segmentation in general, as the boundaries between healthy and infarcted tissue are sometimes not clear-cut, leaving room for interpretation.

In conclusion, our segmentation pipeline is capable of performing on par or, in some cases better, than human experts. Although there are still some weaknesses in MVO segmentation, we have demonstrated that our infarct segmentation algorithm outperforms trained human observers in terms of qualitative segmentation accuracy. If generalizability can be shown on validation on external datasets and readers, our model could be developed for fully automated CMR myocardial infarction quantification in clinical practice.

## Supplementary Material

umaf023_Supplementary_Data

## Data Availability

The data underlying this article will be shared on reasonable request to the corresponding author.
