# Supplementary material for: Deep learning pipeline for fully automated myocardial infarct segmentation from clinical cardiac MR scans
Source: Radiol Adv. 2025 Jul 18;2(4):umaf023. doi: 10.1093/radadv/umaf023 (PMC12429236; doi:10.1093/radadv/umaf023)
Supplement: umaf023_Supplementary_Data [file umaf023_supplementary_data.zip › supplemental_Materials.zip!/supplemental_Materials.pdf]

Supplemental Material

Appendix S1: Dataset Specifics

For all the patients used in our study, the inclusion criteria were: first STEMI according to the ESC/ACC committee criteria (36), revascularization by PPCI within 24 hours after onset of ischemic symptoms, and Killip class <3 at the time of CMR. The following exclusion criteria were applied: age <18 years, TIMI flow 0 and 1 after PPCI, any history of previous myocardial infarction or a non-fatal reinfarction during the study period, an estimated glomerular filtration rate <30 mL/min per 1.73 m^2, and any other contraindication to CMR examination.

All the data used in this study were collected at the Department of Radiology as part of the MARINA-STEMI cohort. For training the algorithm, training and evaluation datasets including examinations between 2006 and 2022 were randomly selected from the cohort. For the test dataset random examinations obtained between 2021 and 2023 were selected.

Appendix S2: Manual Segmentation

Manual segmentations were done in a semiautomated way with the goal of reduceing inter-rater variability. In the first step, the slices with visible myocardial scars were manually selected. On these slices, the epi and endocardial borders were outlined. Then within the myocardium, a region of interest (ROI) was drawn in the non-infarcted myocardial segment opposite to the scar to define the remote myocardium. From the mean signal intensity and standard deviation (SD) of this ROI, a threshold of 5 SDs above the mean signal was chosen. This threshold is based on the literature and our own experience (37), and its goal is to avoid biased assessment of hyperenhanced myocardial regions due to subjective window settings. After windowing with this threshold, the contours of the LGE-enhanced areas were drawn manually. In this manner, the infarct area was quantitatively assessed for each slice and segment. The infarct volume in mL was then calculated by multiplying the hyperenhanced area

by the slice thickness, including the interslice gap. MVO volumes were calculated in the same way, but unlike for LGE segmentations, MVO was labelled on the original images without the use of windowing.

Appendix S3: Imaging Protocol

All images in this study (training, evaluation and test datasets) were acquired on two identical 1.5 Tesla MR scanners from the same vendor (Magnetom Avanto^{Fit}, Siemens, Erlangen, Germany). Each patient underwent LGE CMR imaging after a minimum interval of 10 minutes following the intravenous administration of a gadolinium bolus of 0.2 mmol/kg body weight (Gadobutrol, Gadovist, Schering, Berlin). This procedure employed an ECG-triggered phase-sensitive inversion recovery (PSIR) single-shot TrueFISP sequence with consecutive short-axis slices. Parameters for this scan included a slice thickness of 8 mm, an interslice gap of 2 mm, a FOV of 400 × 363 mm, voxel dimensions of 2.2 × 1.6 × 8.0 mm, a TR of 590 msec, a TE of 1.2 msec, a flip angle of 45°, and a GRAPPA iPat factor of 2.

Appendix S4: Network Architectures and Training Details

For our framework, we trained a total of 3 different networks:

1. A 3D U-net (ROI-net), which is trained to segment the left ventricle in the original clinical MR images.
2. A 2D U-net that is trained to do multiclass segmentation of the left ventricle on a per-slice level.
3. An error-correcting 3D U-Net that improves the 2D segmentation masks for infarction and MVO by including information about neighboring slices.

All the networks were trained on the same training dataset. For the ROI-net, we used the manually marked epicardial boundaries to create binary segmentation masks of LV and

background. Because the original image stacks have different image sizes, we cropped or zero-padded the images to a uniform size of 256 x 256.

For all networks, we use basic U-Net architectures with kernel sizes of 3 x 3 for the 2D network and 3 x 3 x 3 for the 3D networks, respectively (Figure S1). After each convolutional block instance normalization is applied, followed by a leaky rectified linear unit (ReLU) activation function. For the 2D U-Net, downsampling is achieved by max-pooling and upsampling by bilinear interpolation. The 3D CNNs use strided convolutions, following (38), for downsampling as well as strided convolution transposed for upsampling. For the 3D U-Nets, no downsampling or upsampling is performed with respect to the third dimension. ROI-net and the 2D-net have only one input channel. For the final error-correcting 3D CNN, the number of input channels is 3, as the course 2D segmentations of myocardial scar and MVO are added as additional information to the CNN’s input.

ROI-net was trained for 300 epochs on a batch size of 4, and 2D and 3D cascade nets were trained for 750 epochs with batch sizes of 32 and 4, respectively. All models were trained from scratch with randomly initialized network weights. A variation of the Dice loss as proposed in (39) was used as the loss function for all the networks. The loss function was minimized through stochastic gradient descent with Nesterov momentum ($\mu = 0.99$) and an exponentially decaying learning rate. Before AI input, all images were normalized to have a mean of zero and a standard deviation of one. Segmentation performance was evaluated after each training epoch on 33 CMR images from an additional evaluation dataset, which was obtained in the same manner as the training dataset. The best-performing model was then chosen. During training, different data augmentation techniques were applied (Gaussian blurring, gamma correction, additive white noise, changing contrast/brightness, simulating lower resolution, translation, flipping, elastic deformations, scaling). For the cascaded CNN, the perturbation module was switched on only after 100 epochs of training. Furthermore, deep supervision (40) was employed, providing additional direct supervision to some hidden layers in addition to only supervising the output layer. All the training of the framework was performed on a NVIDIA A40 GPU using the Pytorch deep learning library.

Appendix S5: Perturbation Module

In our training pipeline, we utilize an error-correcting 2D-3D cascaded CNN, specifically designed for infarct segmentation and initially proposed in (31). In this framework, first a 2D CNN performs multiclass segmentation on a per-slice level. Then, a 3D CNN corrects the coarse 2D segmentation masks by incorporating information about neighboring slices. However, since the 2D CNN was optimized on the training dataset, its segmentation masks are already quite precise on this data. This fact prevents the subsequent 3D segmentation network from learning to detect and correct 2D segmentation errors during training. The perturbation module, which is interposed between the 2D and 3D networks, addresses the issue of overly precise coarse 2D segmentation masks by incorporating 2D-characteristic errors. More specifically, the module is designed to simulate potential segmentation errors that the first-stage network might encounter due to limited inter-slice information. By artificially generating these errors during training, the subsequent 3D network is enforced to learn how to correct them effectively. The perturbation module introduces several types of artificial segmentation errors (Figure S2):

- **Enhanced data augmentation:** The ranges for contrast, brightness, low-resolution, and gamma augmentations are expanded beyond those used in the 2D augmentation pipeline.
- **Random class deletion:** For certain classes (myocardial scar, MVO, or both), the 2D segmentations are deleted in a single slice of the volume.
- **Infarction misinformation:** Incorrect infarction annotations are added to random slices. This is done by identifying the 85th percentile of pixel values within the myocardium on a chosen slice and adding the largest connected component as a false scar annotation.
- **MVO misinformation:** MVO annotations are falsified by changing the label of a pixel classified as scar, along with some neighboring pixels, to MVO.
- **Complete mask nullification:** In some cases, the entire 2D segmentation mask is set to zero for random samples.

During training, these perturbations are applied with low probabilities (10% for deleting single classes, nullifying the mask, or adding false infarction annotations, and 2% for adding incorrect MVO annotations). Since these perturbations are typically applied to random single slices within a 3D volume, the 3D network is trained to detect and correct errors characteristic of missing inter-slice information, improving the generalizability properties of the final framework on unseen data.

Appendix S6: Qualitative Evaluation

Qualitative evaluation was done on a per-slice level. For each patient, the medical raters could click through all the CMR slices and for each slice they individually assessed subjective segmentation quality. They were able to click back and forth between the raw input image and the segmented image. Without knowing if the segmentation was done manually or automatically, they had to decide for one of the seven segmentation ratings (Figure S3) and type their assessment into an Excel table.

For the comparison between the two methods, both segmentations were presented to the raters side by side. Again, by clicking back and forth, they could display and hide the segmentation masks. Manual and automatic segmentations were distributed randomly between *segmentation 1* and *segmentation 2* such that the raters had no information about which segmentation was done by which method. Comparing the two images, the raters had to decide whether they preferred one of the segmentations or considered them equally good. Again, the rating was conducted for both classes (MVO and scar) and the evaluations were recorded in an Excel sheet.

References

36. Thygesen, K., Alpert, J. S., Jaffe, A. S., Chaitman, B. R., Bax, J. J., Morrow, D. A., White, H. D., & Executive Group on behalf of the Joint European Society of Cardiology (ESC)/American College of Cardiology (ACC)/American Heart Association (AHA)/World

Heart Federation (WHF) Task Force for the Universal Definition of Myocardial Infarction. (2018). Fourth universal definition of myocardial infarction (2018). *Circulation*, *138*(20), e618-e651.

37. Mayr, A., Klug, G., Reindl, M., Lechner, I., Tiller, C., Holzknecht, M., Pamminger, M., Troger, F., Schocke, M., Bauer, A., Reinstadler, S. J., & Metzler, B. (2022). Evolution of myocardial tissue injury: a CMR study over a decade after STEMI. *Cardiovascular Imaging*, *15*(6), 1030-1042.

38. Szegedy, C., Vanhoucke, V., Ioffe, S., Shlens, J., & Wojna, Z. (2016). Rethinking the inception architecture for computer vision. In *Proceedings of the IEEE conference on computer vision and pattern recognition* (pp. 2818-2826).

39. Milletari, F., Navab, N., & Ahmadi, S. A. (2016). V-net: Fully convolutional neural networks for volumetric medical image segmentation. In *2016 fourth international conference on 3D vision (3DV)* (pp. 565-571). IEEE.

40. Lee, C. Y., Xie, S., Gallagher, P., Zhang, Z., & Tu, Z. (2015, February). Deeply-supervised nets. In *Artificial intelligence and statistics* (pp. 562-570). Pmlr.

Supplemental Figures

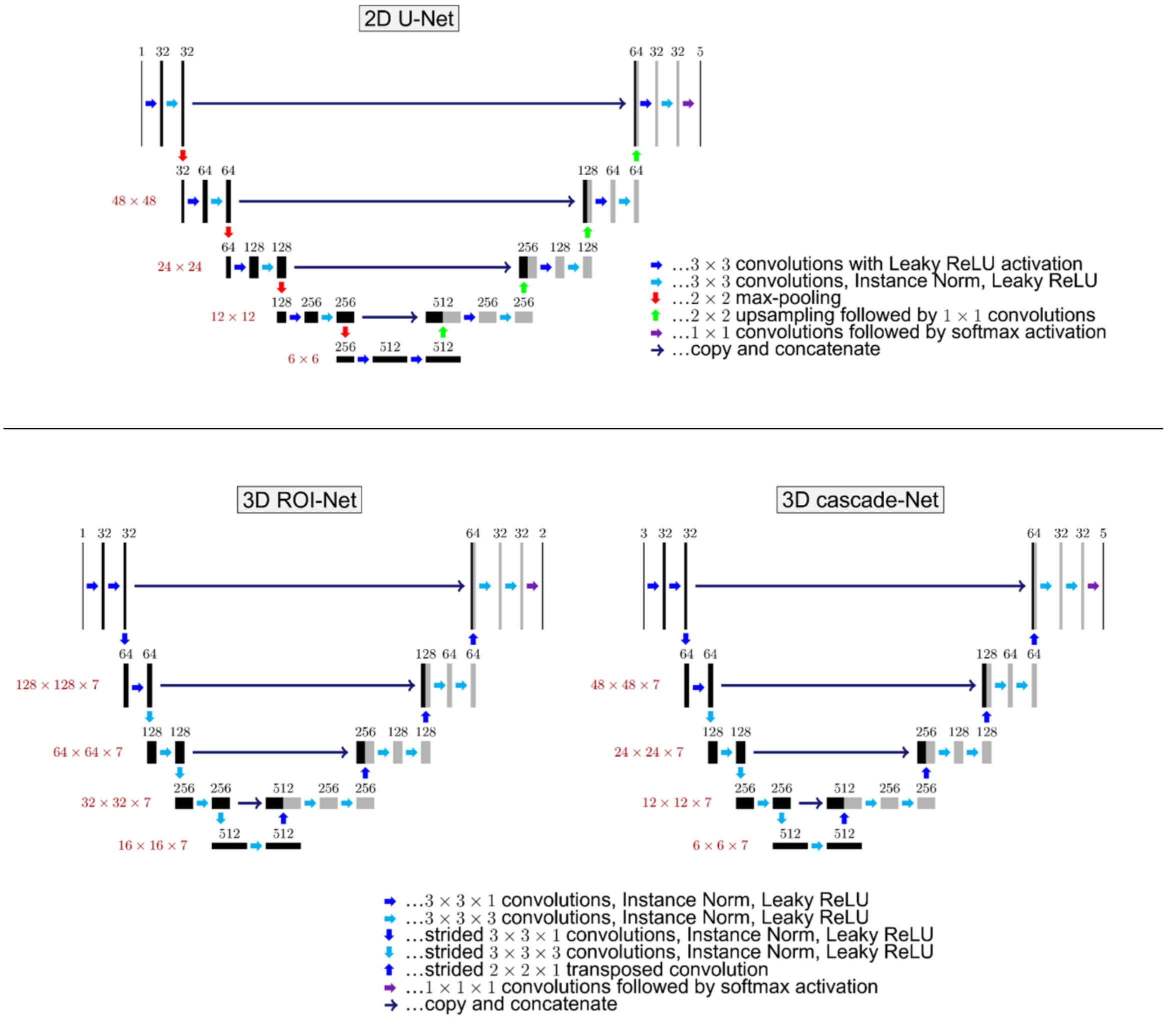

Figure S1: Detailed architectures of the used CNNs.

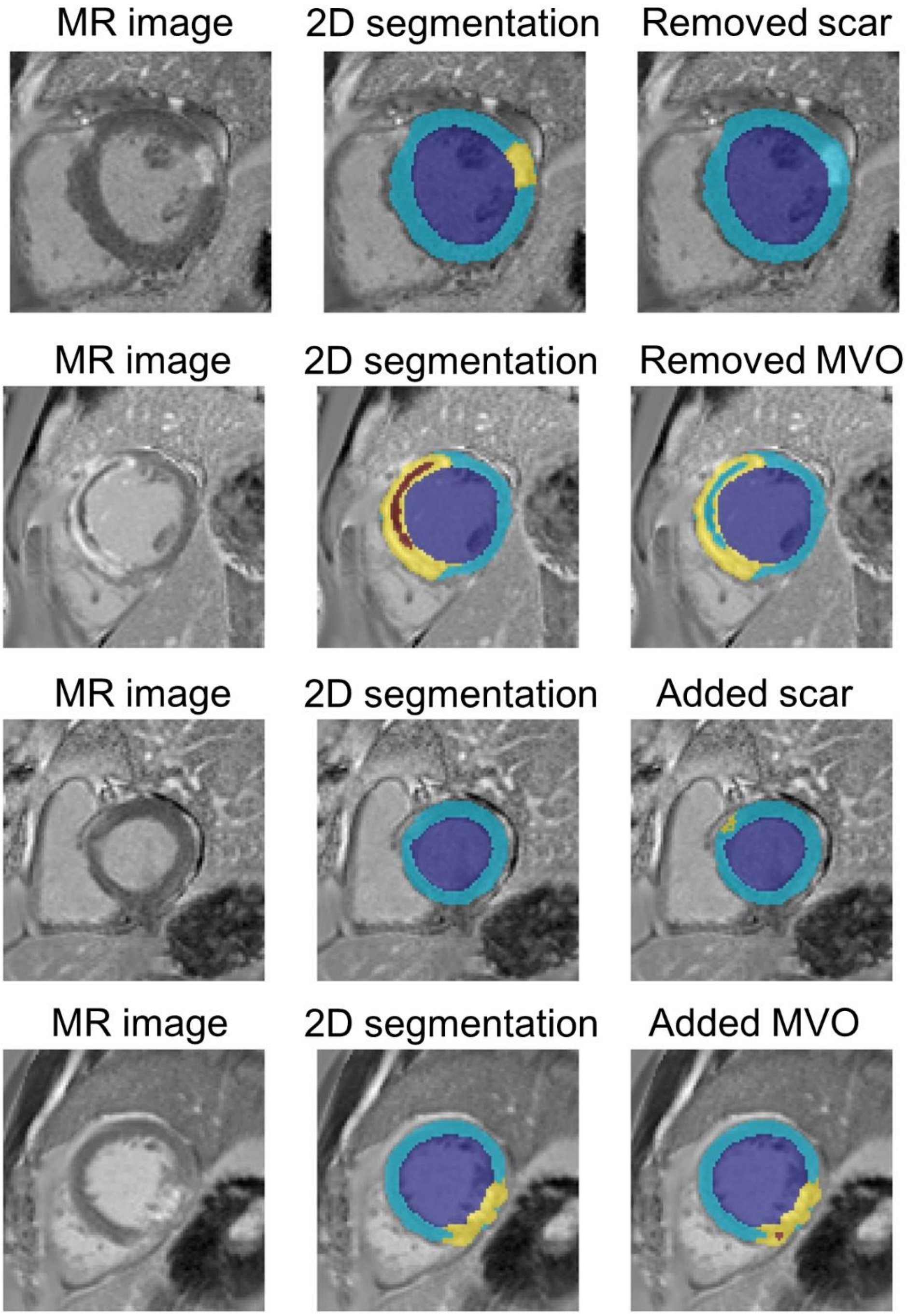

Figure S2: Examples for artificial modifications of the 2D segmentation masks by the perturbation module. The first two lines show examples where correctly identified infarction resp. MVO was removed. The bottom two lines show images where the perturbation module added incorrect segmentations of scar (line 3) and MVO (line 4).

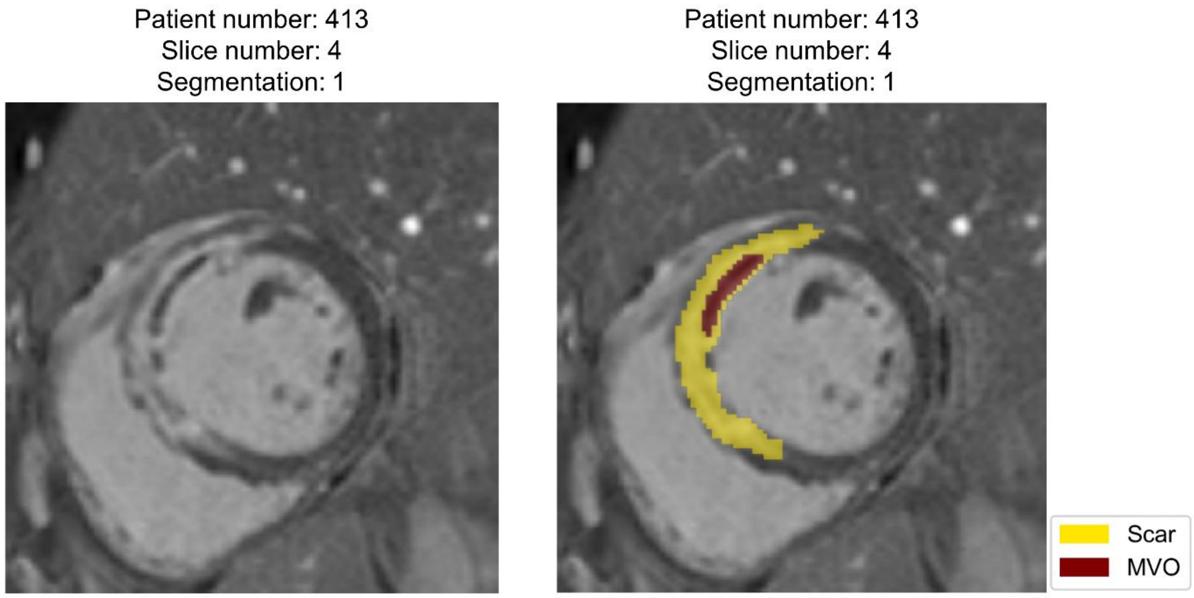

	A	B	C	D	E
1	Pat_numbe	Slice_numbe	Segmentatio	Scar	MVO
2	413	4	1	1	3

0=true negative, 1=optimal, 2=too big, 3=too small, 4=wrong organ, 5=false negative, 6=false positive

Figure S3: Rating process on the individual images. Slice number and anonymized patient number were displayed to the raters. For each image, they could choose between seven different subjective rating categories for both scar and MVO segmentation.

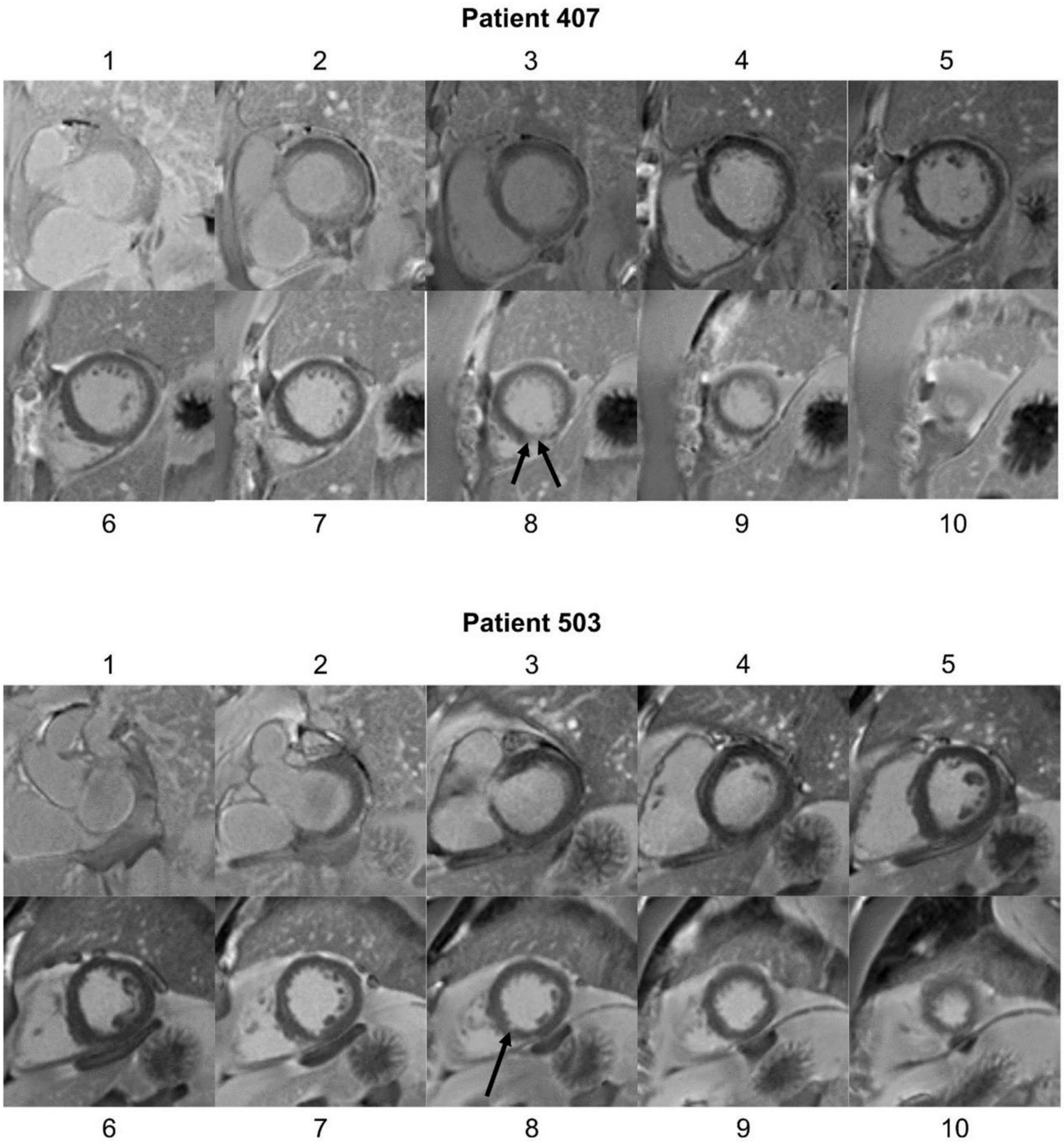

Figure S4: The two patients in whom the proposed method missed the scars. Patient 407 has a very small posterior wall infarct scar apically (Slice 8). Patient 503 has a very hard-to-see scar in slice 8. Both scars are almost impossible to detect in the LGE images and could only be clearly confirmed after additional reviews of the functional images and previous examinations.
